# Evaluation of eating and rumination behaviour in 300 cows of three different breeds using a noseband pressure sensor

**DOI:** 10.1186/s12917-015-0549-8

**Published:** 2015-09-04

**Authors:** Ueli Braun, Susanne Zürcher, Michael Hässig

**Affiliations:** Department of Farm Animals, Vetsuisse-Faculty, University of Zurich, Winterthurerstrasse 260, CH-8057 Zurich, Switzerland

**Keywords:** Cattle, Eating, Rumination, Different breeds, Automated recording

## Abstract

**Background:**

Eating and rumination variables were recorded in 300 healthy lactating dairy cows of 3 different breeds (100 Brown Swiss, 100 Holstein-Friesian, 100 Swiss Fleckvieh cows). Eating and rumination variables were monitored during a 24-h period using an automated system that recorded jaw movements via a pressure sensor integrated into the noseband of a halter. Phases of eating and rumination were reliably identified in the recordings based on typical patterns seen in previous studies. The variables analysed included duration of eating and rumination, number of chewing cycles during eating and rumination, number of regurgitated cuds and number of chewing cycles per cud.

**Results:**

The cows ate for an average of 265 ± 54 min and chewed 17,077 ± 3646 times per day. The duration of rumination was 441 ± 71 min, there were 578 ± 94 cuds per day and 55 ± 10 chewing cycles per cud. There were significant correlations (*P* < 0.01) between duration of eating and number of chewing cycles during eating (*r* = 0.94), between duration of rumination and number of chewing cycles per regurgitated cud (*r* = 0.56) and between duration of rumination and number of regurgitated cuds per day (*r* = 0.53).

**Conclusions:**

The eating and rumination variables established in the present study reflect the current conditions of Swiss dairy farming and serve as reference intervals for assessing sick cows.

## Background

In the past, observation of eating and rumination activities was commonly used by dairy farmers to monitor the health and wellbeing of their cows; some farmers may even have known how long a certain cow chewed her cud. Industrialisation of animal farming has drastically changed this and observation of individual cows has become less important in animal husbandry. Several techniques have been developed for automated recording of eating and rumination activities [1–6]. The technique described by Nydegger and coworkers [6] recorded jaw movements during chewing using a pressure sensor integrated into the noseband of a halter. The eating and rumination behaviour of cows has been studied recently in our clinic. The first study validated pressure sensor recordings by comparing them with direct observation of eating and rumination over a 24-h period in ten cows [7, 8]. The results showed that eating and rumination activities can be easily identified in the recordings and that there is excellent agreement between the automated recordings and direct observation. In another study, eating and rumination behaviour was investigated in cows with various diseases and in the peripartum period [9, 10]. Eating and rumination behaviour on a winter pasture and under loose-housing conditions was compared in Scottish Highland cattle [11]. The objective of the present study was to examine eating and rumination behaviour in 300 dairy cows of different breeds and to establish reference intervals for each breed. An automated system was used to record and analyse eating and rumination variables over a 24-h period in 100 Brown Swiss cows, 100 Holstein Friesian cows and 100 Swiss Fleckvieh cows.

## Methods

### Animals

One hundred Brown Swiss (Brown Swiss x Braunvieh), 100 Holstein Friesian and 100 Swiss Fleckvieh (Simmental x Red Holstein) cows between 2 and 6 years of age (mean ± sd = 3.6 ± 1.35 years) were used (Table 1). The cows were 4 to 20 weeks in milk and the daily production ranged from 25 to 45 kg (31.5 ± 6.97 kg). Each breed group was formed to include 50 cows aged 2 to 4 years (2.5 ± 0.5 years), referred to as young cows, and 50 cows aged 4.1 to 6 years (4.8 ± 0.79 years), referred to as old cows. The young cows produced 29.7 ± 5.5 kg of milk per day and were 10.1 ± 4.75 weeks in milk, and the old cows produced 33.4 ± 7.4 kg of milk per day and were 11.1 ± 5.3 weeks in milk. All cows had been healthy and had not received drugs during the 30-day period before the start of the study.

**Table 1:** Age, milk production and stage of lactation in cows of three breeds and two age groups

Breed	Age group	Age (years) mean ± sd	Milk production (kg/day) mean ± sd	Stage of lactation (weeks) mean ± sd
Brown Swiss (*n* = 100)	young	2.5 ± 0.50	27.4 ± 2.86	8.4 ± 3.53
	old	4.8 ± 0.88	30.3 ± 5.01	10.0 ± 4.54
	combined	3.6 ± 1.35	28.8 ± 4.27	9.2 ± 4.14
Holstein Friesian (*n* = 100)	young	2.5 ± 0.50	32.8 ± 7.13	10.8 ± 4.75
	old	4.9 ± 0.77	36.0 ± 9.05	1.2 ± 5.67
	combined	3.7 ± 1.36	34.4 ± 8.31	12.0 ± 5.37
Swiss Fleckvieh (*n* = 100)	young	2.4 ± 0.49	29.0 ± 4.29	11.1 ± 5.33
	old	4.7 ± 0.78	33.8 ± 6.41	10.2 ± 4.85
	combined	3.6 ± 1.33	31.4 ± 5.96	10.7 ± 5.11
All young cows (*n* = 150)		2.5 ± 0.50	29.7 ± 5.53	10.1 ± 4.75
All old cows (*n* = 150)		4.8 ± 0.79	33.4 ± 7.41	11.1 ± 5.25
All cows (*n* = 300)		3.6 ± 1.35	31.5 ± 6.79	10.6 ± 5.04

### Housing and feeding

The cows originated from 41 dairy farms, which were tie-stall operations with similar management conditions. They had free access to water and were fed hay ad libitum during the day and corn silage/haylage several times a day. Concentrate was fed twice a day according to the level of production. All 41 herds were free of infectious diseases as bovine rhinotracheitis, bovine virus diarrhoea/mucosal disease and tuberculosis.

### Recording protocol

The cows were fitted with the recording halter the night before the examination so they could become accustomed to it. Recording started at 09:00 the next morning and lasted for 24 h. The halter was then removed and the data were analysed.

### Pressure transducer recordings of eating and rumination activities

The recordings were obtained as described recently [6, 8] using a pressure sensor integrated into the noseband of a horse halter (MSR Electronics, Seuzach, Switzerland). The sensor recorded the pressure changes that occurred with each jaw movement. The sensor was connected to a data logger (MSR 145W, MSR Electronics), which was kept in a leather pouch on the side of the halter, and contained a secure digital (SD) card to store the data. The SD card had a 4 GB capacity, which allowed a measuring period of 3 weeks. At the end of one measuring period, the data were uploaded from the logger to a personal computer using the SD card.

### Analysis of pressure transducer recordings

A special software program (R V2.12.1, MSR Electronics) was used to evaluate the data. The analysis was done as recently described [9]. The measured variables included duration of eating and rumination, number of eating and rumination phases, number of chewing cycles during eating and rumination, number of regurgitated cuds and number of chewing cycles per cud.

### Statistical analysis

The STATA 12 software (StataCorp LP, College Station, Texas, USA, 2011) was used for calculating the means, standard deviations and frequency distributions. Sampling was estimated by the following presumptions according to Braun et al. (11): Alpha = 0.05, Power = 0.8, Mean_1_ = 280, Mean_2_ = 250, SD_1/2_ = 50, n_1_/n_2_ = 1, giving n_1_, n_2_ = 99 or 100 as used.

Data were tested for normality using the Wilk-Shapiro test. Oneway analysis of variance (ANOVA) was used to identify significant differences among groups, and differences between groups were analysed using the Bonferroni test. Multivariate ANOVA was used to test for the random effects such as farm. A *P*-value <0.05 was considered significant. Correlation coefficients were calculated for all eating and rumination variables.

### Approval of the study by an ethical committee

The study was approved by The Ethical Committee of the canton of Zurich, Switzerland.

## Results

### Duration of eating

The mean duration of eating for all cows was 265 min (4.4 h) (Table 2). It was significantly longer (*P* < 0.01) in Brown Swiss cows (282 min) than in Holstein Friesian (256 min) and Swiss Fleckvieh cows (258 min). Old cows had a longer duration of eating than young cows (278 versus 253 min, *P* < 0.01).

**Table 2:** Duration of eating in 24 h (minutes, mean ± sd, range in brackets) in 300 cows of three breeds

	Groups		
Breed	All cows (100 per breed)	Young cows, 2 to 4 years (50 per breed)	Old cows, 4.1 to 6 years (50 per breed)
Brown Swiss (*n* = 100)	282 ± 56^a^ (226–338)	271 ± 54 (217–326)	293 ± 55 (237–348)
Holstein Friesian (*n* = 100)	256 ± 50 (206–307)	248 ± 47 (202–295)	264 ± 53 (211–318)
Swiss Fleckvieh (*n* = 100)	258 ± 51 (207–309)	239 ± 44 (195–283)	276 ± 51 (225–327)
All cows (*n* = 300)	265 ± 54 (211–319)	253 ± 50^b^ (203–303)	278 ± 55 (223–332)

^a^Compared with other breeds *P* < 0.01

^b^Compared with old cows *P* < 0.01

### Chewing cycles during eating

The cows had on average 17,077 chewing cycles related to eating (Table 3). Brown Swiss cows (18,120 cycles) had significantly more chewing cycles than Holstein Friesian (16,522 cycles) and Swiss Fleckvieh cows (16,588 cycles). Young cows had significantly fewer chewing cycles (16,525 cycles, *P* < 0.01) than old cows (17,628 cycles).

**Table 3:** Number of chewing cycles during eating in 24 h (mean ± sd, range in brackets) in 300 cows of three breeds

	Groups		
Breed	All cows (100 per breed)	Young cows, 2 to 4 years (50 per breed)	Old cows, 4.1 to 6 years (50 per breed)
Brown Swiss (*n* = 100)	18,120 ± 3830^a^ (14,290–21,950)	17,566 ± 3850 (13,716–21,416)	18,674 ± 3728 (14,946–22,402)
Holstein Friesian (*n* = 100)	16,522 ± 3528 (12,994–20,050)	16,204 ± 3462 (12,742–19,667)	16,840 ± 3564 (13,276–20,404)
Swiss Fleckvieh (*n* = 100)	16,588 ± 3335 (13,252–19,923)	15,804 ± 3158 (12,646–18,961)	17,372 ± 3324 (14,047–20,696)
All cows (*n* = 300)	17,077 ± 3646 (13,431–20,722)	16,525 ± 3582^b^ (12,943–20,106)	17,628 ± 3626 (14,003–21,254)

^a^Compared with other breeds *P* < 0.01

^b^Compared with old cows *P* < 0.01

### Duration of rumination

The mean duration of rumination for all cows was 441 min (7.35 h) (Table 4). It was significantly shorter (*P* < 0.01) in Brown Swiss cows (405 min) than in Holstein Friesian (458 min) and Swiss Fleckvieh cows (460 min). The duration of rumination did not differ between young and old cows.

**Table 4:** Duration of rumination in 24 h (minutes, mean ± sd, range in brackets) in 300 cows of three breeds

	Groups		
Breed	All cows (100 per breed)	Young cows, 2 to 4 years (50 per breed)	Old cows, 4.1 to 6 years (50 per breed)
Brown Swiss (*n* = 100)	405 ± 64^a^ (341–469)	409 ± 53 (355–462)	401 ± 73 (328–474)
Holstein Friesian (*n* = 100)	458 ± 73 (385–530)	453 ± 77 (376–529)	463 ± 68 (395–531)
Swiss Fleckvieh (*n* = 100)	460 ± 60 (400–520)	462 ± 61 (401–523)	458 ± 58 (400–517)
All cows (*n* = 300)	441 ± 71 (370–511)	441 ± 68 (373–509)	440 ± 73 (368–513)

^a^Compared with other breeds *P* < 0.01

### Number of regurgitated cuds

The mean number of cuds for all cows was 578 (Table 5). There were significant differences among the breeds (*P* < 0.01); Swiss Fleckvieh cows had the largest number (624) followed by Holstein Friesians (589) and Brown Swiss cows (522). The number of cuds did not differ between young and old cows.

**Table 5:** Number of regurgitated cuds in 24 h (mean ± sd, range in brackets) in 300 cows of three breeds

	Groups		
Breed	All cows (100 per breed)	Young cows, 2 to 4 years (50 per breed)	Old cows, 4.1 to 6 years (50 per breed)
Brown Swiss (*n* = 100)	522 ± 75^a^ (448–597)	543 ± 70 (473–612)	502 ± 74 (428–757)
Holstein Friesian (*n* = 100)	589 ± 81^a^ (508–669)	591 ± 87 (503–678)	587 ± 73 (513–660)
Red Holstein (*n* = 100)	624 ± 96^a^ (528–720)	635 ± 84 (551–720)	623 ± 70 (553–693)
All cows (*n* = 300)	578 ± 94 (484–672)	586 ± 99 (487–685)	570 ± 88 (482–658)

^a^Difference among all breeds *P* < 0.01

### Number of chewing cycles per cud

The mean number of chewing cycles per cud for all cows was 55 (Table 6). There were no differences among the three breeds with respect to this variable.

**Table 6:** Number of chewing cycles per cud (mean ± sd, range in brackets) in 300 cows of three breeds

	Groups		
Breed	All cows (100 per breed)	Young cows, 2 to 4 years (50 per breed)	Old cows, 4.1 to 6 years (50 per breed)
Brown Swiss (*n* = 100)	55 ± 9 (46–64)	56 ± 9 (47–65)	55 ± 9 (46–64)
Holstein Friesian (*n* = 100)	56 ± 11 (45–67)	57 ± 13 (44–70)	55 ± 8 (47–63)
Swiss Fleckvieh (*n* = 100)	53 ± 9 (45–62)	55 ± 9 (46–64)	52 ± 8 (44–59)
All cows (*n* = 300)	55 ± 10 (45–65)	56 ± 10 (46–66)	54 ± 9 (45–62)

### Farm effect on eating and rumination variables

There was a farm effect on duration of eating (*P* < 0.01), number of chewing cycles during eating (*P* < 0.05) and number of chewing cycles per cud (*P* < 0.01) but not on duration of rumination (*P* > 0.05) and the number of regurgitated cuds (*P* > 0.05).

### Correlations between eating and rumination variables

There were significant correlations (*P* < 0.01) between duration of eating and number of chewing cycles during eating (*r* = 0.94), between duration of rumination and number of chewing cycles per cud (*r* = 0.56) and between duration of rumination and number of regurgitated cuds per day (*r* = 0.53).

## Discussion

The mean duration of eating during a 24-h period was 265 min (211 to 319 min). Brown Swiss cows ate significantly longer than Holstein Friesian and Swiss Fleckvieh cows, and older cows ate longer than younger cows regardless of breed. This result was in agreement with published daily eating times of 198 to 264 min [12], 220 to 303 min [13], 248 to 292 min [14], 301 min [15] and 330 min [16]. One study reported a shorter time of 185 to 214 min [17], and in other studies, the upper limit was considerably higher than in the present study: 240 to 420 min [18], 462 min [19], 375 to 497 min [8], 240 to 540 min [20]. The variation in published reference intervals is related to various factors that affect feed intake including breed, age, body weight and milk production of the cow, type of ration and feeding management, energy requirements, environmental factors and social structure of the herd [21]. In our own study [8]), that yielded relatively long eating times, the cows were offered fresh hay day and night and the long eating times confirmed the close relationship between the availability of fresh feed and feed intake [22, 23]. In the present study, the cows were fed routinely from early morning until evening, but not during the night.

Farm was considered a random variable because it is affected by management, composition of the ration as well as the content of fibre and energy and major and trace elements.

The cows had on average 17,077 chewing cycles related to eating per day, fewer than values recorded by other authors: 18,766 cycles [15], 21,629 cycles [19], 24,000 cycles [20]. As expected, the number of chewing cycles was strongly (*r* = 0.94) and positively correlated with the duration of eating.

The mean rumination time was 441 min with a range of 370 to 511 min. This was in general agreement with published daily rumination times of 240 to 540 [24], 300 to 540 [18], 413 to 454 [17], 441 to 479 [7, 8], 457 [15], 462 [19], 464 to 579 [14] and 498 to 584 min [12]. One study had a range of 459 to 621 min and an upper limit that exceeded ours by more than 100 min [13]. There are several factors that affect duration of rumination, but the most important is the physical structure of the feed. Small feed particles are associated with shorter rumination times [25–27]. Restrictive feeding leads to faster eating and swallowing of larger feed particles and in turn to longer rumination times [18]. Stress [28], anxiety [11] and illness [29–31] lower rumination activity. The percentage of cows that ruminate at a certain time has been used to assess rumen health in dairy cows [12, 32, 33].

The mean number of regurgitated cuds was 578 ± 94 (484 to 672) per day, which was in general agreement with published ranges for healthy cows of 348 to 478 [7], 360 to 790 [20] and 366 to 611 cuds [9].

The number of chewing cycles per cud (55 ± 10, 45 to 65) had quite a large variation. Ranges observed in other studies were 40 to 70 [24], 43 to 69 [7] and 53 to 57 chewing cycles per cud [9]. Cows fed a ration with larger forage particles may respond by increasing the duration of eating and rumination or by increasing the number of chewing cycles per cud [26]. The number of chewing cycles also increases with increasing fibre content of the ration [18].

As discussed previously, feed intake is subject to many factors and varies among herds. The results of the present study should therefore be interpreted as guidelines for eating and rumination variables. However, this is the first study of this kind in a large number of cows, which improves the reliability of the presented data, although the findings do not apply universally. Eating and rumination characteristics are sensitive indicators of many disorders including postpartum metabolic diseases, and we recommend that large herds establish separate normal ranges when major ration changes occur. In experimental studies, it is critical to include a control group that is kept under identical conditions as the studied group. The intra-individual daily variations of eating and rumination variables are crucial for the assessment of a cow’s health status, analogous to the monitoring of daily milk yield. A sudden drop in eating and rumination activity in an individual cows indicates illness but also can be related to the onset of parturition [10]. When multiple cows are affected, external factors should be considered. For instance, when a sudden drop in eating and rumination variables was observed in a small herd of Scottish Highland cattle, prompt investigation of the problem showed a defective drinking water supply, which had caused the cows to go off feed [11].

## Conclusions

The eating and rumination variables established in the present study are based on the observation of 300 cows from modern dairy herds, reflect the current conditions of Swiss dairy farming and serve as reference intervals for detecting sick cows.
